# Pulmonary rehabilitation, physical activity and aortic stiffness in COPD

**DOI:** 10.1186/s12931-019-1135-6

**Published:** 2019-07-24

**Authors:** Yousef S. Aldabayan, Heidi A. Ridsdale, Ahmed M. Alrajeh, Abdulelah M. Aldhahir, Arthur Lemson, Jaber S. Alqahtani, Jeremy S. Brown, John R. Hurst

**Affiliations:** 1 UCL Respiratory, University College London, London, UK; 2 Central and North West London NHS Foundation Trust, London, UK; 3 Radboud University, Nijmegen, NL Netherlands

**Keywords:** COPD, Aortic stiffness, And pulmonary rehabilitation

## Abstract

**Background:**

Patients with chronic obstructive pulmonary disease (COPD) have elevated cardiovascular risk, and cardiovascular disease is a major cause of death in COPD. The current literature indicates that changes in cardiovascular risk during pulmonary rehabilitation (assessed using aortic stiffness) are heterogeneous suggesting that there may be sub-groups of patients who do and do not benefit.

**Objectives:**

To investigate the characteristics of COPD patients who do and do not experience aortic stiffness reduction during pulmonary rehabilitation, examine how changes relate to physical activity and exercise capacity, and assess whether changes in aortic stiffness are maintained at 6 weeks following rehabilitation.

**Methods:**

We prospectively measured arterial stiffness (aortic pulse-wave velocity), exercise capacity (Incremental Shuttle Walk Test) and physical activity (daily step count) in 92 COPD patients who started a six week pulmonary rehabilitation programme, 54 of whom completed rehabilitation, and 29 of whom were re-assessed six weeks later.

**Results:**

Whilst on average there was no influence of pulmonary rehabilitation on aortic stiffness (pre- vs. post pulse-wave velocity 11.3 vs. 11.1 m/s *p* = 0.34), 56% patients responded with a significant reduction in aortic stiffness. Change in aortic stiffness (absolute and/or percentage) during rehabilitation was associated with both increased physical activity (rho = − 0.30, *p* = 0.042) and change in exercise capacity (rho = − 0.32, *p* = 0.02), but in multivariable analysis most closely with physical activity. 92% of the responders who attended maintained this response six weeks later.

**Conclusion:**

Elevated aortic stiffness in COPD is potentially modifiable in a subgroup of patients during pulmonary rehabilitation and is associated with increased physical activity.

**Trial registration:**

ClinicalTrials.gov Identifier: NCT03003208. Registered 26/12/ 2016.

## Background

Chronic obstructive pulmonary disease (COPD) is a leading cause of global morbidity and mortality [1]. Patients with COPD have more co-morbidity than smoking-matched controls [2], including a 2–5 fold greater risk of cardiovascular (CV) disease [3]. CV diseases are a major cause of death in COPD [4]. As reported by the World Health Organisation, COPD will be the third most common cause of death by 2030 if no new interventions are put in place [5], and reducing mortality in COPD requires health-care professionals to take an holistic approach. Early detection and prediction of cardiovascular (CV) risk is therefore critical in people with COPD [4]. Arterial stiffness assessed by aortic Pulse Wave Velocity (aPWV) is recognised as a gold-standard biomarker of increased CV risk in COPD, as it is in healthy populations [6–8]. Elevated arterial stiffness occurs as a consequence of biological aging and atherosclerosis which may lead to increased risk of cardiovascular mortality [9]. It has been consistently reported that the main contributing factors relating to increased arterial stiffness are hypertension, metabolic disorders and chronic inflammation [10] all of which are commonly present in COPD [11]. Therefore, reducing aortic stiffness may lower the risk of future CVD which is a major cause of death in COPD. However, it remains un-known whether increased aortic stiffness in COPD is modifiable [12, 13]. We have previously reported that CV risk in COPD is stable over time, but elevated at exacerbations [14].

It is known that physical activity (PA) reduces CV risk. In coronary artery disease, exercise programmes reduce aPWV [15]. Reduced physical activity is common in COPD [16, 17]. Pulmonary Rehabilitation (PR), a group exercise and education programme is an evidence-based intervention in COPD to reduce symptoms, improve exercise performance, reduce exacerbations and improve health-status [18–21]. We have previously reviewed the literature on the effect of PR on aortic stiffness in COPD [22]. Whilst the large and well-conducted study by Vanfleteren reported that, on average, there was no influence of PR on arterial stiffness in COPD, the data suggest that arterial stiffness responses to PR were highly heterogeneous such that there may have been sub-groups of patients who did and did not benefit. Previous work has not examined the relationship between physical activity, PR outcomes and aortic stiffness in COPD. We hypothesised that patients who had the greatest physical activity, and the greatest improvement in exercise capacity during PR would be those that experienced the greatest aortic stiffness reduction. This study aimed to investigate the characteristics of COPD patients who do and do not experience aortic stiffness reduction during PR. We also wanted to examine how changes in aPWV relate to physical activity and exercise capacity and assess whether changes in aortic stiffness during PR were sustainable 6 weeks after the end of the class.

## Method

### Participants

**Fig. 1:**
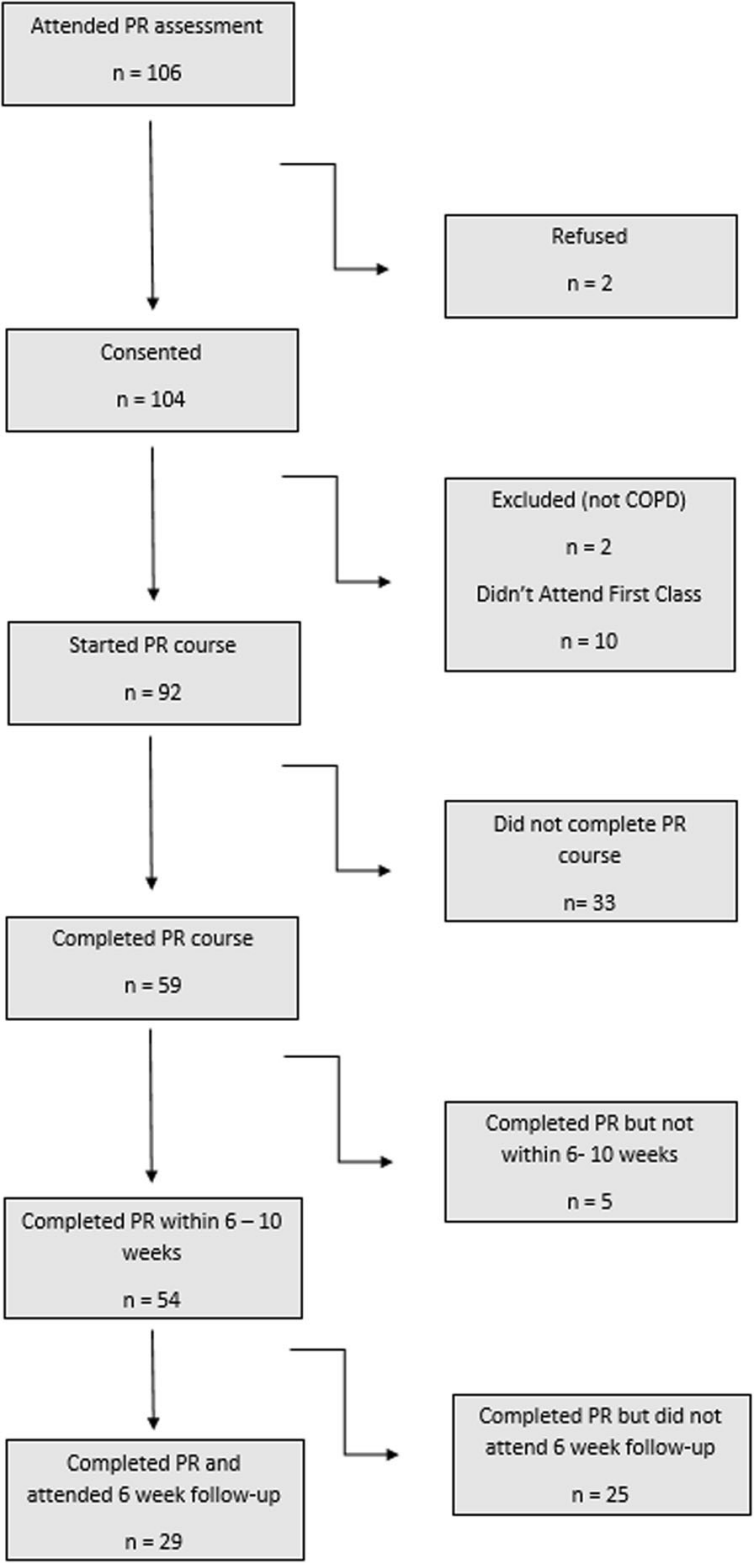
Consort diagram

We approached 106 consecutive patients enrolling on the Central and North West London NHS Foundation Trust PR classes held at the Peckwater Center, and St. Pancras Hospital in London, UK. A total of 102 (58 male, 44 female) with a confirmed diagnosis of COPD (post-bronchodilator FEV1/ FVC < 0.70 and appropriate exposure history) were recruited (Fig. 1; only two patients refused to take part). Participants referred to PR were scheduled for an assessment visit before the first class, performed by registered physiotherapists. In patients agreeing to take part, a full medical history including cardiovascular risk and co-morbidities was documented during this assessment visit. Of 102 participants, 54 completed PR with complete pre- and post-measurements and this group comprised the main analysis.

### Pulmonary rehabilitation

The PR course consisted of sessions two hours long, twice each week for six weeks. The PR programme is based on British Thoracic Society (BTS) PR guidelines [23]. The first hour comprised an exercise component to both lower and upper limbs supervised by respiratory physiotherapists. It included low resistance training exercises such as free weights, and therabands. It also included aerobic exercises such as treadmill, walking, and cycling. The intensity of the workout was individualised based on the condition of each participant. Physiotherapists encouraged the participants to exercise for a minimum of 10 min on each exercise at level 3 to 4 on the Borg scale. The second hour consisted of education which was delivered by a multidisciplinary team including nurses, physiotherapists, doctors, psychologists, dietitians, and occupational therapists.

### Measurements

Comprehensive data were gathered from each participant including demographic and clinical information. COPD exacerbation frequency was defined as the number of events treated with oral antibiotics and or corticosteroids in the previous year. Breathlessness, quality of life, and anxiety & depression were assessed using CAT COPD [24], MRC dyspnoea [25] and HADS [26] questionnaires respectively. Aortic stiffness was directly measured by arterial pulse wave velocity (aPWV; further detail below) and calculated using QRISK2 to estimate the risk of having a heart attack or stroke over the next ten years [27].

The following measurements were made at the start and end of PR, and the duration between these times and number of classes attended were recorded.

### Arterial stiffness measurements (aPWV)

Arterial stiffness was determined by measuring aPWV between the carotid and femoral arteries using Vicorder (Skidmore Medical, Bristol, UK) equipment. Based on the manufacturer’s instructions, the participant was asked to lie at 45°. Then, both of the carotid and femoral cuffs were attached. The carotid cuff was positioned over the carotid palpation area and the femoral cuff was positioned around the upper right thigh. Next, the participant lay supine to measure the distance between the suprasternal notch and each of the femoral and carotid arteries. After the distance was recorded, the bed was raised back to 45° to start the aPWV measurement, expressed in meters per second. After 10 min rest, one aPWV set was measured for each participant (three readings a set). The mean of those measures was used. We have previously reported that aPWV is stable over time in patients with COPD [14] and thus any changes that we see during PR can likely be attributed to the PR intervention.

### Spirometry measurements (FEV_1_, FVC and FEV_1_/FVC)

Participants were referred to PR with spirometry results confirming COPD. However, to ensure contemporaneous lung function results, we performed post-bronchodilator hand-held spirometry using a Micro 1 Handheld Spirometer (CareFusion, Basingstoke, UK) which conform to the requirements of the ATS/ERS standards [28] and these values were used for analysis. The participants were seated during this test. Measurements were made in triplicate to published quality-assurance criteria [29].

### Exercise capacity: incremental shuttle walking test (ISWT)

The ISWT was conducted in accordance to the ERS/ATS guidelines [30]. Participants were instructed in how to perform the test. Two cones were placed a distance of 9 m apart. Next, a pre-recorded CD played a dictated tempo from a metronome such that walking speed was externally paced. The number of laps and time given are divided into 12 levels, each level containing an additional lap compared to the previous one and the shortened time between laps requires increased walking speed. Heart rate, oxygen saturation and dyspnea (Borg scale) were measured prior to and directly after the test. These outcomes were re-measured 1 and 2 min after recovery. The walking test was terminated when the patient was unable complete a full shuttle within the time frame allowed. To counteract a possible learning effect and ensure a maximal result on the ISWT, we conducted a second ISWT. The test showing the higher distance was used in the analysis.

### Physical activity monitoring

We asked participants to wear a step counter pedometer on their waist all the time whether inside or outside the PR class (except when sleeping and showering) and to record the total daily physical activity on a diary card. This was for the six-week duration of the class and for the subsequent six-weeks after PR completion. We used a Yamax SW-200 electronic pedometer, which has previously been shown to be a reliable and valid device [31–33]. One week after the start of PR we contacted patients to make sure that they were able to use and record data from the step counter.

### Follow-up

Participants who completed the PR programme were asked to attend again six weeks after the end of PR for re-evaluation. Exercise was not supervised during this period; however, physical activity was monitored by providing a step counter pedometer. At the end of the six weeks, patients were re-assessed, completing the same evaluations described above.

### Statistical analysis

Data were assessed using histograms and tested for normality using the Kolmogorov–Smirnov test. Data are expressed as mean (SD) for normally distributed data or median (IQR) for non-normally distributed data as appropriate. We examined the change in aPWV pre- and post-exercise and classified our participants as responders (reduction of ≥0.5 m/s) or non-responders (less benefit than this).

To complete a power calculation we contacted the authors of the largest previous study [13], who reported that 35% of their participants were responders according to these criteria (≥0.5 m/s reduction). A priori, and as described in the trial registration, we planned a multivariable analysis on responder status including change in exercise capacity (change in ISWT pre- and post), physical activity (mean of steps/day) with or without inclusion of one other variable decided on the basis of significance in simple correlation analysis. This required us to have 30 responders. For other comparisons, paired t-tests were used for parametric data, and Wilcoxon signed-rank testing was used for non-parametric paired data. Relationships were analysed using Pearson correlation for normally distributed data, and Spearman rank correlation for non-parametric data. Data was analysed using Statistical Package for the Social Sciences (SPSS), Version 21.

## Results

### Baseline characteristics of the patients

We approached 106 consecutive patients enrolling on two PR programmes in Camden, London, UK and 104 agreed to take part. Spirometry did not confirm COPD in two of these. The CONSORT diagram is illustrated as Fig. 1. Ultimately, 92 patients started and 54 patients completed PR within 6–10 weeks of starting (termed “completers” and this group forms the main analysis). The characteristics of these 54 patients are provided in Table 1, which shows they had a mean age of 73 years, 63% were male and the mean FEV_1_ was 1.23 L (50% predicted). Table 1 also provides information on the 102 total population, and compares the 54 completers with the 48 non-completers (who attended the assessment visit but did not start, or complete PR within the designated time). The dropout rate during PR was 36%. The completers were generally similar to the non-completers in age and sex, but tended to have more severe COPD and greater baseline aortic stiffness.

**Table 1:** Baseline characteristics of subjects with chronic obstructive pulmonary disease (COPD) referred to PR and consented for the study, divided into those who did and did not complete PR

Subjects Demographics	Total population (102)	Completed PR (54)	Not completed (48)	*p*-value
Age (years)	71.31 ± 9.06	72.71 ± 8.48	69.71 ± 9.54	0.10
Male	59 (58%)	34 (63%)	25 (52%)	0.27
Female	43 (42%)	20 (37%)	23 (48%)	
Active smoker	30 (29%)	12 (22%)	18 (37%)	0.09
Ex-smokers	72 (71%)	42 (78%)	30 (63%)	
Smoking history (pack-years)	45 (27–63)	47 (23–60)	44 (31–66)	0.49
Body composition				
Body Mass Index (kg/m^2^)	26.70 ± 6.09	26.47 ± 6.13	26.99 ± 6.10	0.67
Pulmonary function				
FEV1 (L)	1.26 ± .41	1.23 ± 0.41	1.51 ± 0.58	0.01
FEV1 (% predicted)	50.69 ± 16.29	50.47 ± 17.55	60.29 ± 19.39	0.01
FEV1/FVC %	47.51 ± 11.88	49.20 ± 12.28	58.07 ± 10.93	< 0.01
Haemodynamic				
Aortic pulse wave velocity (m/s)	10.84 ± 2.29	11.34 ± 2.33	10.05 ± 2.03	0.01
Systolic pressure (mmHg)	138.75 ± 18.33	139.91 ± 19.04	137.29 ± 17.76	0.47
Diastolic pressure (mmHg)	80.15 ± 13.48	80.38 ± 14.13	79.98 ± 12.97	0.91
Mean arterial pressure (mmHg)	98.68 ± 15.94	100.19 ± 12.98	96.93 ± 18.85	0.31
Pulse pressure (mmHg)	77.70 ± 14.59	76.94 ± 16.91	78.73 ± 11.545	0.52
Functional outcomes (pre-PR)				
ISWT (m)	254.32 ± 118.41	254.1 ± 116.6	254.6 ± 122.1	0.98
mMRC grade	3 (2–4)	3 (2–4)	3 (2–4)	0.65
CAT	19.96 ± 8.06	18.72 ± 6.72	21.56 ± 8.78	0.08
Anxiety scores (HADS)	5 (2–7)	5 (2–7)	6 (3–11)	0.06
Depression scores (HADS)	5 (2–6)	5 (2–6)	6 (4–8)	0.06
CV risks determinants				
Diabetes	Yes: 18 (18%)	Yes: 7 (13%)	Yes: 9 (19%)	0.37
Hypertension	Yes: 54 (54%)	Yes: 29 (46%)	Yes: 25 (52%)	0.56
Hyperlipidaemia	Yes: 44 (43%)	Yes: 24 (44%)	Yes: 20 (42%)	0.78
Ischemic heart disease	Yes: 5 (5%)	Yes: 1 (2%)	Yes: 4 (8%)	^a^
Myocardial infarction	Yes: 4 (4%)	Yes: 3 (6%)	Yes: 1 (2%)	^a^
Peripheral arterial disease	Yes: 7 (7%)	Yes: 4 (7%)	Yes: 3 (6%)	^a^
Heart failure	Yes: 7 (7%)	Yes: 2 (4%)	Yes: 5 (10%)	^a^
Atrial fibrillation	Yes: 13 (12%)	Yes: 9 (17%)	Yes: 4 (8%)	^a^
Stroke	Yes: 10 (10%)	Yes: 3 (6%)	Yes: 7 (15%)	^a^

Data are presented as mean (SD), median (IQR) or n (%) as appropriate. *FEV*_*1*_ forced expiratory volume in 1 s, *FVC* forced vital capacity, *ISWT* incremental shuttle walk test, *mMRC* modified Medical Research Council, *HADS* hospital anxiety and depression score.^a^ too few for comparison

### Effectiveness of PR

First, we wanted to confirm that the PR programme met standard goals. In the 54 completers there was a significant improvement from baseline to completion in mean (SD) ISWT (254.3 ± 118.4 vs. 305.1 ± 115.0 m, *p* < 0.001), CAT questionnaire (18.7 ± 6.7 vs. 16.4 ± 6.7, *p* < 0.01) and mMRC dyspnoea score (3(2–4) vs. 3(2–3), *p* < 0.001).

### Primary analysis

**Fig. 2:**
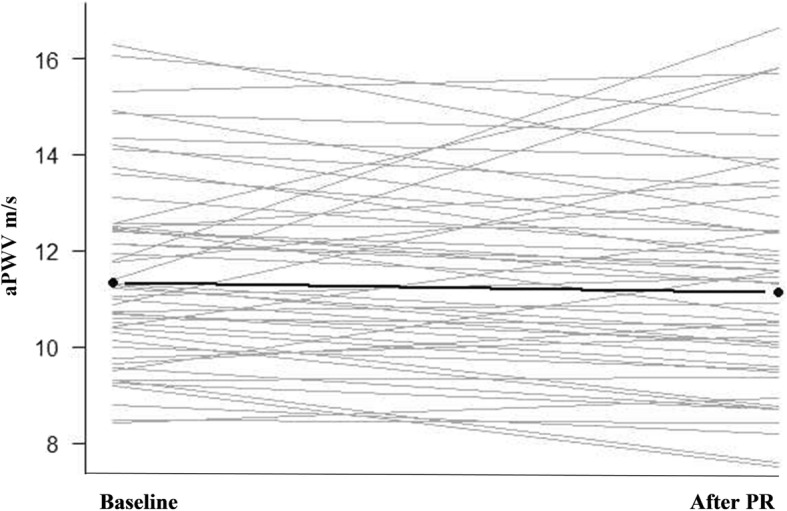
Individual changes in aortic pulse wave velocity (aPWV) before and after pulmonary rehabilitation. Solid line and circles represent the mean

As expected, we did not see an overall difference in aortic stiffness in response to PR (Table 2; Fig. 2). There was a trend to an overall reduction in systolic and mean arterial blood pressure, that was not statistically significant but at a clinically meaningful level (> 3 mmHg).

**Table 2:** Differences in aortic pulse wave velocity and other haemodynamics measures in 54 COPD subjects who completed PR

Haemodynamic measurement	Baseline	After PR	*p*-value
Aortic pulse wave velocity (m/s)	11.34 ± 2.33	11.14 ± 2.58	0.34
Systolic pressure (mmHg)	139.91 ± 19.04	135.84 ± 14.51	0.09
Diastolic pressure (mmHg)	80.38 ± 14.13	78.91 ± 10.914	0.39
Mean arterial pressure (mmHg)	100.19 ± 12.98	96.07 ± 16.75	0.09
Pulse pressure (mmHg)	76.94 ± 16.91	78.15 ± 12.31	0.62

As previously reported by Vanfleteren [13], we did identify a group of individuals who experienced a clinically significant change in aPWV in response to PR. Of the 54 patients, 30 (56%) had a significant response (defined as a reduction of 0.5 m/s or greater) and 24 had no clinically significant improvement.

As described above, in our a priori statistical plan, we had elected to perform a multi-variable analysis including change in ISWT, average step count over the duration of the PR class, and the possibility of a third variable chosen if simple correlation of other variables against change in PWV proved to be statistically significant. However, in simple correlation analysis, we did not identify any other factors associated with change in PWV after PR (Table 3). The step count data during PR is included in Table 4.

**Table 3:** Correlation between change in aortic stiffness (aPWV) after PR with baseline demographic and clinical measures in 54 patients with COPD

Outcomes	Mean ± SD, or median (IQR)		*p*-value
Age (years)	72.71 ± 8.48	*r* = − 0.01	0.93
FEV_1_ (L)	1.23 ± 0.41	*r* = −0.10	0.47
FEV_1_ (% predicted)	50.47 ± 17.55	*r* = −0.09	0.49
FEV_1_/FVC %	49.20 ± 12.28	*r* = 0.05	0.71
BMI (kg/m^2^)	26.47 ± 6.13	*r* = 0.02	0.85
SBP (mmHg)	139.97 ± 18.87	*r* = 0.21	0.12
DBP (mmHg)	80.30 ± 14.01	*r* = 0.11	0.42
MAP (mmHg)	96.07 ± 16.75	*r* = 0.18	0.19
QRISK%	32.23 ± 16.87	*r* = −0.02	0.86
Smoking (pack years)	47 (23–60)	*r* = − 0.08	0.56

**Table 4:** Differences in aortic pulse wave velocity, physical activity and exercise capacity among all patients, responders, and non-responders between pre, the end of PR and six weeks later

All participants						Responders					Non-responders					
Outcomes	Pre PR (*n* = 54)	Post PR (*n* = 54)	At 6 weeks (*n* = 29)	P^a^	P^b^	Pre PR (*n* = 30)	Post PR (*n* = 30)	At 6 weeks (*n* = 13)	P^a^	P^b^	pre PR (*n* = 24)	Post PR (*n* = 24)	At 6 weeks (*n* = 16)	P^a^	P^b^	P^c^
aPWV m/s	11.34 ± 2.33	11.14 ± 2.58	10.98 ± 2.62	0.34	0.37	11.58 ± 2.43	10.44 ± 2.20	10.16 ± 2.61	< 0.001	0.76	11.04 ± 2.20	12.01 ± 2.80	11.66 ± 2.49	0.01	0.17	0.05
ISWT m	254.1 ± 116.64	305.1 ± 115.0	299.0 ± 99.0	< 0.001	0.86	263.6 ± 121.0	322.5 ± 120.4	332.1 ± 99.8	< 0.001	0.55	242.1 ± 112.3	283.3 ± 106.3	270.0 ± 91.2	< 0.001	0.77	0.10
Outcomes	During PR (*n* = 54)		During 6 weeks following PR (*n* = 29)		P^d^	During PR (*n* = 30)		During 6 weeks following PR (*n* = 13)		P^d^	During PR (*n* = 24)		During 6 weeks following PR (*n* = 16)		P^d^	P^c^
PA step/day	4376 ± 2719		4228 ± 2314		0.19	5235 ± 2723		5576 ± 2021		0.38	3355 ± 2390		3255 ± 2044		0.37	0.01

Data are presented as mean (SD). *aPWV* aortic Pulse Wave Velocity, *ISWT* incremental shuttle walk test, *PA* average physical activity

^a^ = pre versus post; ^b^ post versus six weeks; ^c^ responders versus non-responders at the six week time point; ^d^ during PR versus during follow up

Next, we went on to explore the relationship between change in aPWV and the two variables we had specifically intended to examine: change in exercise capacity (assessed by ISWT) and physical activity (assessed using average step count).

**Fig. 3:**
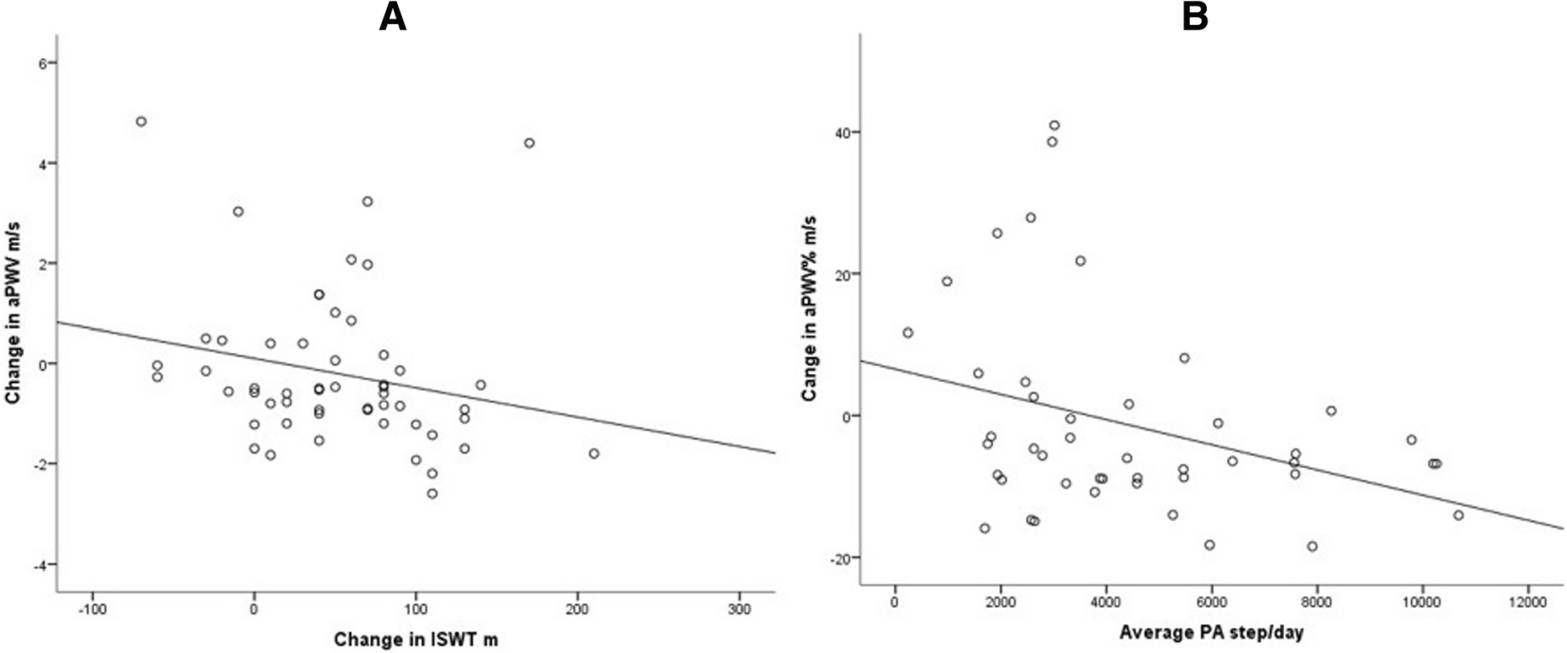
Scatter plots demonstrating correlations between absolute ∆aPWV and ∆ISWT after completing PR (**a** – rho = − 0.32, *p* = 0.020) and between ∆aPWV% and average PA during the six weeks PR (**b** – rho = − 0.30, *p* = 0.042)

Change in aPWV related to both change in exercise capacity and to physical activity. In simple correlation analysis, there was a significant association between larger reduction in aPWV and greater change in ISWT (rho = − 0.32, *p* = 0.020, Fig. 3a) and, when expressed as percentage change, there was a significant association between greater reduction in PWV and higher physical activity (rho = − 0.30, *p* = 0.042, Fig. 3b).

We noted that the starting blood pressure was higher in the responders than the non-responders (103.61 ± 12.55 vs. 95.90 ± 12.44 mmHg, *p* = 0.03) and that a greater proportion of the non-responders were already prescribed anti-hypertensive medication (81% vs. 39%, p = 0.02). The blood pressure in the responders reduced by a clinically and statistically significant degree: the mean fall in systolic BP was 6.30 mmHg (*p* = 0.043), diastolic BP was 4.94 mmHg (*p* = 0.041) and mean arterial pressure was 5.39 mmHg (*p* = 0.020). There were no alterations in any of the participant’s medications during the rehabilitation programme.

We performed multivariable regression analysis to better understand the relationship between physical activity, exercise capacity and change in aortic stiffness during PR. For every 1000 additional steps walked during PR, adjusted for baseline aPWV, aPWV decreased by 0.2 m/s (95%CI, 0.4 to 0.0 m/s change, *p* = 0.03). There was not an independent effect of change in exercise capacity on pulse wave reduction (*p* = 0.19).

### Follow-up

Next we wanted to assess whether changes in aortic stiffness, exercise capacity and physical activity were maintained six weeks after the end of the PR class. The results for the 29/54 patients in whom these data were available are presented in Table 4. There were no overall differences in PWV or exercise capacity comparing the end of PR with six weeks later, or average physical activity during PR compared to physical activity in the following six weeks.

We specifically wanted to examine whether those patients who experienced aortic stiffness reduction with PR maintained this response at six weeks. The mean aPWV was not different in the responders from the end of PR to the six week visit (10.44 vs. 10.16 m/s, *p* = 0.76), and of the 13 people in this group only one patient experienced a subsequent rise in PWV of ≥0.5 m/s. The beneficial aortic stiffness reduction was therefore maintained in 92% of the responders who attended. Non-responders included those who had a change in aPWV within 0.5 m/s of baseline (the “no change” group) and those who had a > 0.5 m/s increase (the “increased” group) during PR. We subdivided our non-responders and showed that the “no change” group maintained similar aortic stiffness (10.79 vs. 10.82 m/s, *p* = 0.86) and exercise capacity (314 vs. 291 m, *p* = 0.18) at six weeks following PR completion. In contrast, the six patients who appeared to have an increase in aPWV during the PR programme showed a significant reduction in aPWV at six weeks (13.43 vs. 12.86 m/s, *p* < 0.001), associated with a significant increase in exercise capacity during this period (208 vs. 235 m, *p* = 0.02), perhaps suggesting a delayed response to PR in this group.

Finally, we wanted to assess over this second six-week period whether PA and change in exercise capacity still related to change in aPWV. In simple correlation analysis aPWV related to both physical activity (rho = − 0.55, *p* = 0.03) and change in exercise capacity (rho = − 0.61, *p* < 0.01) suggesting that patients who maintain activity and gains in exercise capacity are those who maintain the aortic stiffness response achieved during PR.

## Discussion

We report that elevated aortic stiffness in COPD can be reduced in 56% of patients through effective pulmonary rehabilitation, and that this reduction is correlated with greater physical activity and a reduction in blood pressure. Moreover, in the majority of patients who have a reduction in aortic stiffness, this improvement in aortic stiffness reduction is maintained six weeks after the end of PR. This has not been previously reported: existing studies indicated that aortic stiffness was not influenced by PR overall and were not able to identify the characteristics of responder and non-responder groups, and nor was the durability of the response assessed. Our results widen the accepted benefits of PR to include reduction of aortic stiffness, suggesting that PR programmes should focus on maximising physical activity outside of the course to achieve holistic benefits, and demonstrating that elevated aortic stiffness in COPD is durably modifiable with PR.

56% of patients completing PR in our study had a significant reduction in aortic stiffness (defined as a 0.5 m/s reduction or greater). The responders had a clinically meaningful and statistically significant reduction in blood pressure. Change in aPWV was related to both change in exercise capacity during PR, and to physical activity outside of the class. Multivariable analysis confirmed that physical activity was the more important of these two factors. Six weeks later, 92% of the responders who attended maintained this benefit, with both average physical activity and change in exercise capacity in the six weeks following PR related to change in aortic stiffness over this period. Change in aortic stiffness was not restricted to those with or without a previous history of cardiovascular diseases or recognised cardiac risk factors. There was no alteration in potential confounders such as anti-hypertensive medication during PR. Our results emphasise the importance of physical activity on reduction of aortic stiffness in COPD.

We have recently published a systematic review summarising studies examining the effect of PR on CV risk reduction measured by aPWV in COPD [22]. This identified three papers. Our results are in keeping with the largest of these by Vanfleteren [13], a well-conducted study which found no overall difference in aPWV with PR, but in which there were a group of patients who responded. Vanfleteren was not able to identify the reasons for this differential response, but did not measure physical activity – our major hypothesis. Our response rate at 56% is higher than the 35% response rate in this study (personal communication). The two smaller studies identified in our review [34, 35] both found an overall reduction in aortic stiffness with exercise. Vivodtzev investigated the effect of endurance training on aortic stiffness in 17 patients with COPD. There was a 10% reduction in arterial stiffness after exercise endurance in the trained COPD group [34]. Gale investigated the impact of pulmonary rehabilitation on reducing aortic stiffness in COPD. In a study of 32 COPD patients and 20 healthy controls, there was a significant improvement in aortic stiffness in COPD patients after PR [35]. Since publication of our original review we have identified one further study. Moore evaluated the relationships between exercise capacity, physical activity and cardiovascular risk in COPD patients during PR. Once again, there was no overall change in arterial stiffness with PR but by grouping participants into low- and high-exercise tolerance groups, they reported that the lower exercise tolerance group had a better CV benefit [36]. Physical activity was not related to reduction in cardiovascular risk in this study, but activity was only measured for three days prior to commencing PR. Importantly, our study is the only one to measure physical activity during PR, and the only one to re-assess patients six weeks following PR to assess if benefits obtained during PR are maintained.

People with COPD are known to be less active than healthy controls [37]. Physical inactivity is a major risk factor for CV disease in general, and a predictor of mortality in COPD [38]. Reduced physical activity is associated with increased cardiovascular risk and may potentially explain the increased prevalence of CV diseases in people with COPD [16]. A systematic review [17] demonstrated consistent associations between physical activity, mortality and exacerbations, but found insufficient evidence to make firm conclusions about the major determinants of physical activity in COPD. COPD is often accompanied by hypertension and the American Heart Association have stated that increasing physical activity reduces blood pressure and therefore may reduce cardiovascular risk [39]. Given the impact of physical activity on adverse outcomes in COPD, improving physical activity should be a key goal of COPD management. There is little previous information on the relationship between exercise capacity and aortic stiffness in COPD. Stickland reported that VO_2_ max during a cardiopulmonary exercise testing test was independently associated with arterial stiffness in COPD subjects without CVD [37].

In our study, 56% of patients had a beneficial aortic stiffness response during PR, defined as a reduction of 0.5 m/s or greater in aPWV. Meta-analysis of > 12 000 subjects has demonstrated that a difference of ≥0.5 m/ s in aPWV corresponds to a 7.5% reduction in CV risk, such that a 0.5 m/s reduction in aPWV can be considered clinically meaningful [3]. We therefore selected a reduction of 0.5 m/s or greater as a clinically meaningful change in aPWV. Walking an additional 2500 steps was associated with a reduction of 0.5 m/s in aPWV. However, walking an additional 2500 steps/day might be a challenge for patients with more severe COPD.

The question of whether elevated aortic stiffness is modifiable in COPD is clinically important. Our study builds on studies suggesting that CV risk in COPD may be modifiable with respiratory interventions such as long-acting bronchodilators and inhaled corticosteroids [40], and not solely require interventions directly targeting the CV system.

The strengths of this study include the fact that patients with and without CV history were approached sequentially, and therefore that our subjects are representative of those attending PR classes in the UK. The completers were similar to the non-completers, except that they had slightly elevated aortic stiffness and COPD severity. All testing was performed to established guidelines and quality assurance, and arterial stiffness was determined by aPWV which is a gold standard method for measuring arterial stiffness. Our study was adequately powered, with an a priori power calculation and statistical plan. One of the limitations of our study is the relatively high dropout rate (36%). However, this is a recognised problem in pulmonary rehabilitation programmes and our rate is lower than the UK national COPD Audit data reporting a 38% dropout rate [41]. The reasons for high attrition rates in PR and how to mitigate this remain poorly understood and require further study. PR in other settings may be of a different, often longer duration, and this may be a more effective approach. Similarly, not all participants attended the six week follow-up appointment after the six week PR. We measured physical activity using a pedometer. We selected the best available device and deliberately chose simple, inexpensive equipment such that our results can be implemented easily in clinical practice. We recognise that additional information on physical activity and intensity would have been provided using an accelerometer, and that such equipment does not rely on participants manually recording and resetting the device on a daily basis. However, steps are a more intuitive concept for patients and clinicians to understand than markers of energy expenditure, and the chosen Yamax pedometer has been identified as the most reliable and valid device available [31–33]. Finally, we only assessed the durability of the response in aortic stiffness out to six weeks post-PR, and future studies would usefully consider longer term responses.

A previous study in our department [14] recruiting a similar cohort (*n* = 90, age 72.07 ± 9.92 years and FEV_1_ 1.28 ± 0.54 L) monitored aortic stiffness in COPD patients over time in the absence of PR. In this study the proportion of participants who had a decrease in aortic stiffness of > 0.5 m/s was 30% compared to 56% in our study with the PR intervention. In our current study, non-responders were defined as those who did not achieve > 0.5 m/s reduction in aortic stiffness. This included those with no change (26%; aPWV remained within 0.5 m/s of baseline) and those with greater than a 0.5 m/s increase in aortic stiffness. Among the 90 patients in the previous study with no PR intervention, the proportion of those with an increase of more than 0.5 m/s was 23/90 (26%) compared to 10/54 (18%) of our participants who attended PR. This indicates a downward shift in the whole population in aPWV in patients undergoing PR compared to those assessed over time with no intervention.

Our results have important implications for clinical practice. First, they emphasise the value of encouraging people with COPD to maintain physical activity, and that encouraging this during PR may be associated with optimal, holistic benefits from PR in reducing aortic stiffness. Second, our study increases the evidence base for PR in that we have shown that PR can reduce aortic stiffness in a proportion of patients with COPD, likely through an effect on reducing blood pressure. Importantly this benefit was not restricted to those with (or without) established CV disease, and we provide the first evidence that a non-pharmacological COPD-targeted intervention may be able to modify aortic stiffness in COPD for at least six weeks.

## Conclusions

We report that elevated aortic stiffness in COPD is potentially modifiable in a subgroup of patients through pulmonary rehabilitation and that this reduction is related to physical activity. This improvement was maintained for at least six weeks following PR. Our results widen the accepted benefits of PR to include reduction of aortic stiffness, suggest that PR programmes should focus on maximising physical activity, and demonstrate that elevated aortic stiffness in COPD is modifiable with PR.

## Data Availability

Any dataset or resources used in the current study may be available from the corresponding author on reasonable request.

## Abbreviations

aPWV: Aortic pulse wave velocity
BMI: Body max index
BTS: British Thoracic Society
CNWL: Central and North West London
COPD: Chronic obstructive pulmonary disease
CV: Cardiovascular
CVD: Cardiovascular disease
DBP: Diastolic blood pressure
FEV_1_: Forced expiratory volume in 1 second
FVC: Forced vital capacity
HADS: Hospital anxiety and depression score
ISWT: Incremental Shuttle Walking Test
MAP: Mean arterial pressure
mMRC: Modified Medical Research Council
PA: Physical activity
PR: Pulmonary rehabilitation
SBP: Systolic blood pressure
SPSS: Statistical Package for the Social Sciences
UCL: University College London
